# Ethyl Rosmarinate Prevents the Impairment of Vascular Function and Morphological Changes in L-NAME-Induced Hypertensive Rats

**DOI:** 10.3390/medicina55120777

**Published:** 2019-12-07

**Authors:** Rungusa Pantan, Jiraporn Tocharus, Archawin Nakaew, Apichart Suksamrarn, Chainarong Tocharus

**Affiliations:** 1 Department of Anatomy, Faculty of Medicine, Chiang Mai University, Chiang Mai 50200, Thailand; rungusa.p@gmail.com; 2 Department of Physiology, Faculty of Medicine, Chiang Mai University, Chiang Mai 50200, Thailand; 3 Department of Chemistry and Center of Excellence for Innovation in Chemistry, Faculty of Science, Ramkhamhaeng University, Bangkok 10240, Thailand

**Keywords:** hypertension, vascular function, ethyl rosmarinate, endothelial dysfunction

## Abstract

*Background and Objectives:* The potent, endothelium-independent, vasorelaxant effect of ethyl rosmarinate, an ester derivative of rosmarinic acid, makes it of interest as an alternative therapeutic agent for use in hypertension. This study was designed to investigate the effect of ethyl rosmarinate on N^ω^-nitro-L-arginine methyl ester (L-NAME)-induced hypertensive rats. *Materials and Methods:* L-NAME was given orally to male Wistar rats for 6 weeks to induce hypertension concurrently with treatment of ethyl rosmarinate at 5, 15, or 30 mg/kgor enalapril at 10 mg/kg Systolic blood pressure (SBP), heart rate, and body weight of all experimental groups were recorded weekly, while the vascular sensitivity and histological changes of the aorta were evaluated at the end of the experiment. *Results:* For all treatment groups, the data indicated that ethyl rosmarinate significantly attenuated the SBP in hypertensive rats induced by L-NAME, with no significant differences in heart rate and body weight. In addition, the response of vascular sensitivity to acetylcholine (ACh) was improved but there was no significant difference in the response to sodium nitroprusside (SNP). Furthermore, the sensitivity of the aorta to phenylephrine (PE) was significantly decreased. The thickness of the aortic wall did not differ between groups but the expression of endothelial nitric oxide synthase (eNOS) was increased in ethyl rosmarinate- and enalapril-treated groups compared with the hypertensive group. *Conclusions:* Ethyl rosmarinate is an interesting candidate as an alternative treatment for hypertension due to its ability to improve vascular function and to increase the expression of eNOS similar to enalapril which is a drug commonly used in hypertension.

## 1. Introduction

It is well established that the impact of the progression of hypertension and other cardiovascular disease is associated with the impairment of vascular endothelial cell integrity, known as endothelial dysfunction [1,2]. It is characterized by the imbalance of vasodilators and contracting factors, which leads to an impairment of endothelium-dependent vasodilation. Nitric oxide (NO), which is synthesized from L-arginine and oxygen catalyzed by the enzyme nitric oxide synthase (NOS), is important in blood pressure control. In the vascular wall, endothelial cells have an essential role in balancing NO production, which affects vascular smooth muscle cells in the regulation of vascular tone, via both direct and indirect mechanisms [3,4]. There is a large body of evidence to support that the chronic inhibition of NO synthase produces high blood pressure and is related to phenotypic change and proliferation of vascular smooth muscle cells (VSMC) [5,6]. Furthermore, many in vivo studies have demonstrated that chronic inhibition of NO production by Nω-nitro-L-arginine methyl ester (L-NAME) in rats causes a significant increase in blood pressure and also enhances vascular wall thickening [7,8,9]. Consequently, diagnosis and treatment that improve the function of vascular endothelial cells would reduce the risk and rate of death from cardiovascular diseases.

Rosmarinic acid is a phenolic compound found in various plants species, including *Rosmarinus officinalis* and *Hyptis suaveolens*, and shows many beneficial effects on health. These include anti-inflammatory, anti-oxidative, hepatoprotective, antiviral, and antibacterial effects [10,11,12,13,14,15]. A previous study reported that rosmarinic acid showed vasodilatory activity mediated through an endothelium-dependent pathway [16]. However, the vasorelaxant activity of this natural compound is low. Subsequently, a number of ester derivatives of rosmarinic acid were synthesized and their vasorelaxant activity was evaluated. Ethyl rosmarinate is one of the ester derivatives of rosmarinic acid that has been shown to have greater vasodilatory activity in the rat aorta. The mechanisms involved in the regulation of vasodilation by ethyl rosmarinate occur through an endothelium-independent pathway. These mechanisms involve the opening of voltage-gated potassium (K_v_) channels and the blockade of intracellular and extracellular Ca^2+^ channels [17]. These advantageous properties of ethyl rosmarinate are suitable for hypertensive patient who lose their endothelial function. However, there is no evidence of anti-hypertensive effect of ethyl rosmarinate. We therefore decided to investigate the effect of ethyl rosmarinate on hypertension induced by L-NAME in rats, which contributes to establish a new alternative anti-hypertensive drug. The alteration of blood pressure, vascular function, and morphological changes in the aorta were assessed. 

## 2. Materials and Methods

### 2.1. Preparation of Ethyl Rosmarinate

Rosmarinic acid (2.0 g) was dissolved in absolute ethanol (40 mL) and concentrated sulfuric acid (1 mL) was added. The mixture was stirred overnight and water (100 mL) was then added. The mixture was extracted with ethyl acetate where the organic layer was rinsed with water and dried over anhydrous sodium sulfate before evaporation. The crude product was purified using silica column chromatography by elution with ethyl acetate-methanol (100:1) to yield ethyl rosmarinate (5 g). The spectroscopic (NMR and mass spectra) data were consistent with those reported previously [18].

### 2.2. Animals

Male Wistar rats (200–250 g), obtained from the National Laboratory Animal Center, Mahidol University, Salaya (Nakornpathom, Thailand), were housed in separated cages under standard laboratory conditions in a 12 h light/dark cycle at room temperature (25 ± 2 °C) with free access to rodent diet and water. The use of animals conformed to the international and national guidelines for ethical conduct on the care and use of animals, which was approved by the animal ethics committee of the Faculty of Medicine, Chiang Mai University, Thailand (approval number: 1/2556; approval date: 22 May 2013).

### 2.3. Effect of Ethyl Rosmarinate on Blood Pressure, Heart Rate, and Body Weight 

Rats were divided into seven groups of six animals each: (1) a control group (CTRL), where rats were given tap water; (2) control rats plus ethyl rosmarinate (CTRL + ER), where rats were supplemented with 30 mg/kg of ethyl rosmarinate; (3) hypertensive rats (HT), where rats were given 40 mg/kg of L-NAME dissolved in tap water; (4) three groups of hypertensive rats plus different concentrations of ethyl rosmarinate (HT + ER), including rats that simultaneously received 40 mg/kg of L-NAME and 5, 15, or 30 mg/kg of ethyl rosmarinate; and, the final group, (5) hypertensive rats plus enalapril (HT + Enal) in which rats received, simultaneously, 40 mg/kg of L-NAME and 10 mg/kg of enalapril. Ethyl rosmarinate and enalapril were given by oral gavage. Blood pressure and heart rate were measured using the tail-cuff method (ADInstruments, Sydney, Australia). The alteration in systolic blood pressure (SBP), heart rate, and body weight were recorded weekly for 6 weeks. At the end of experiment, rats were sacrificed and the thoracic aorta was excised in each case to enable vascular function, histopathology, and immunohistochemistry assessments.

### 2.4. Effect of Ethyl Rosmarinate on Vascular Reactivity 

The adherent connective tissue and fat were cleaned from the isolated aorta which was then cut into rings approximately 3 mm in length. Following this, the rings were mounted on tungsten wire and immersed in a chamber bath, containing normal physiological Krebs solution (composition, mM: NaCl, 122; KCl, 4.9; 4-(2-hydroxyethyl)-1-piperazineethanesulfonic acid (HEPES), 10; KH_2_PO_4_ 0.5; NaH_2_PO_4_, 0.5; MgCl_2_, 1.0; glucose, 11.0; and CaCl_2_, 1.8. pH 7.3), maintained at 37 °C, and oxygen was bubbled continuously through the solution. The system was connected to FT-104 isometric force transducers (Iworx System, Inc., Dover, NH, USA), which measured the response of the aortic rings through the PowerLab data acquisition system (ADIntruments, Sydney, Australia) and recorded using the Lab Chart 7 Software program (ADInstruments, Sydney, Australia). The optimal resting tension of 1 g was initially applied to the aortic ring and equilibrated for 1 h. Each test of vascular reactivity was started pre-contraction with 1 μM of phenylephrine (PE). To investigate the possible effects of ethyl rosmarinate amelioration on the effect of L-NAME-inhibited vasodilation, the cumulative concentration-response curves of acetylcholine (ACh) (from 3 × 10^−12^ to 3 × 10^−5^ M) and sodium nitroprusside (SNP; from 3 × 10^−12^ to 3 × 10^−6^ M) were constructed. Additionally, the cumulative concentration-response curves of PE (from 3 × 10^−9^ to 3 × 10^−6^ M) were constructed to determine the role of ethyl rosmarinate in α_1_-adrenergic receptor-regulated vasoconstriction. 

### 2.5. Effect of Ethyl Rosmarinate on Morphological Changse of the Aorta 

To evaluate the morphological changes in the aorta, the aorta was immediately fixed with 4% paraformaldehyde for 48 h. After that, the sample was embedded in paraffin, which was then sliced in cross-section using a microtome and stained with hematoxylin and eosin (H&E). Finally, the stained sections were observed under a light microscope (Olympus BX51; Olympus Co., Tokyo, Japan).

### 2.6. Effect of Ethyl Rosmarinate on the Expression of eNOS

To determine the effect of ethyl rosmarinate on the presence of endothelial nitric oxide synthase (eNOS), immunohistochemistry was investigated. The aortic sections were deparaffinized and rehydrated with xylene and a decreasing gradient of ethanol solutions (100–70%). Then, an antigen-retrieval procedure was performed by heating the sections with citric acid buffer. After that, the endogenous peroxidase activity and non-specific antigens were blocked with 0.3% h×ydrogen peroxide (H2O2) and normal goat serum, respectively. Then, the aortic sections were incubated with the primary antibody of eNOS (Millipore Corporation, Burlington, MI, USA) overnight at 4 °C before being incubated with second antibody (anti-rabbit IgG antibody; Millipore Corporation, MI, Burlington, USA) for 30 min. Finally, the sections were incubated with 3,3-diaminobenzidine tetrahydrochloride (DAB) and counterstained with hematoxylin. The presence of dark brown staining was detected under a 400× power microscope (AX70 Olympus, Olympus Co., Tokyo, Japan).

### 2.7. Effect of Ethyl Rosmarinate on Vasorelaxation Associated with eNOS Pathway

To elucidate the mechanism of ethyl rosmarinate-regulated vasodilation related to the eNOS pathway, the endothelium-intact aortic rings were pre-incubated with 100 µM of L-NAME or L-NAME plus 100 µM of ethyl rosmarinate (ER + L-NAME) before pre-contraction by 1 µM of PE. Then, the rings were induced into vasorelaxation using ACh (10^−10^-10^−5^) cumulatively. The results were compared between those with and without L-NAME.

### 2.8. Statistical Analysis

All data were expressed as mean ± SEM, which were analyzed using a one-way analysis of variance (ANOVA) followed by Dunnett’s post hoc test. A value of *p* < 0.05 was considered significant. The response curves and experimental data were plotted using GraphPad Prism 5 (GraphPad Prism Software Inc., San Diego, CA, USA).

## 3. Results

### 3.1. Chronic Effects of Ethyl Rosmarinate on SBP, Heart Rate, and Body Weight

**Figure 1:**
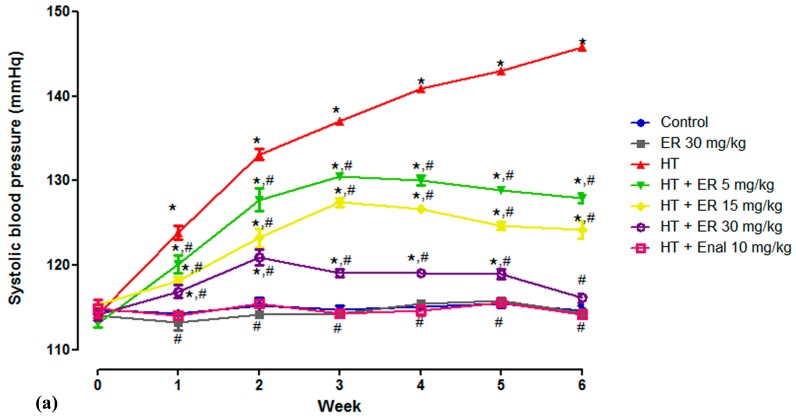

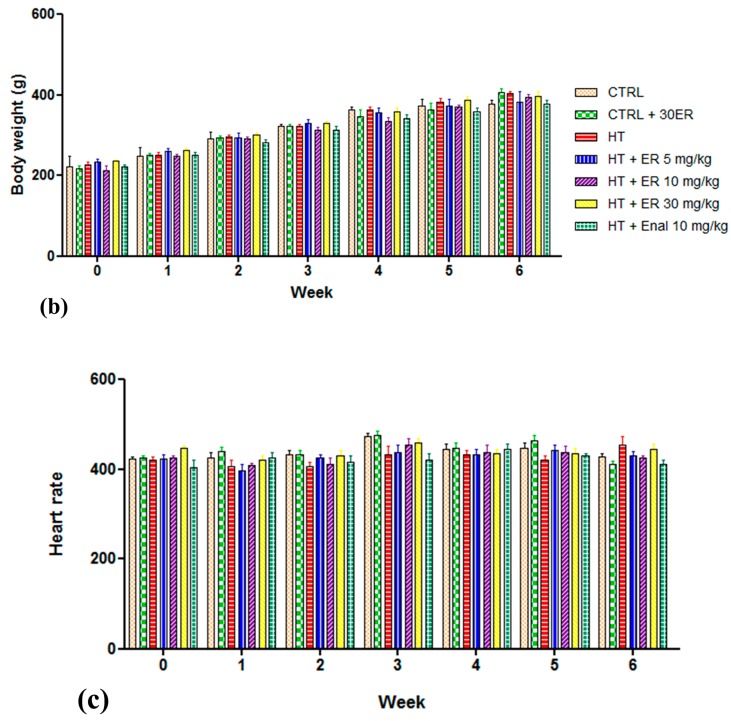
The alteration in (**a**) systolic blood pressure, (**b**) heart rate, and (**c**) body weight recorded weekly from the control group (CTRL), the control group plus 30 mg/ kg ethyl rosmarinate (CTRL + ER 30 mg/kg), hypertension group (HT), hypertension group plus 5, 15, or 30 mg/kg ethyl rosmarinate (HT + ER 5, 15, 30 mg/kg), and the hypertension group plus enalapril 10 mg/kg (HT + Enal 10 mg/kg). Data are expressed as mean ± SEM. *p < 0.05 vs. control group and ^#^*p* < 0.05 vs. hypertension group.

At baseline, there was no significant difference in SBP among the experimental groups. After the first week of daily oral administration of L-NAME at 40 mg/kg, SBP was significantly increased. In the HT group, SBP increased to 145.78 ± 0.32 mmHg at the end of the experiment, which was statistically significantly different to the CTRL group with an SBP of 114.67 ± 0.53 mmHg. However, the SBP in animals treated concomitantly with L-NAME and ethyl rosmarinate at 5, 15, or 30 mg/kg was significantly less than that in the HT group with readings of 127.94 ± 0.55, 124.17 ± 1.05, and 116.11 ± 0.56 mmHg, respectively. In addition, the SBP was fully attenuated by enalapril at 10 mg/kg, with an SBP of 114.16 ± 0.28 mmHg, as shown in Table 1 and Figure 1. In addition, the result showed that 30 mg/kg of ethyl rosmarinate alone did not alter the SBP of the normal rats.

Likewise, heart rate and body weight were recorded every week. The study showed that heart rate and body weight did not differ significantly between the groups.

### 3.2. Ethyl Rosmarinate Attenuated Hypertension-Induced Endothelial Dysfunction

#### 3.2.1. Effect of Ethyl Rosmarinate on Endothelium-Dependent Induced Vasorelaxation

**Figure 2:**
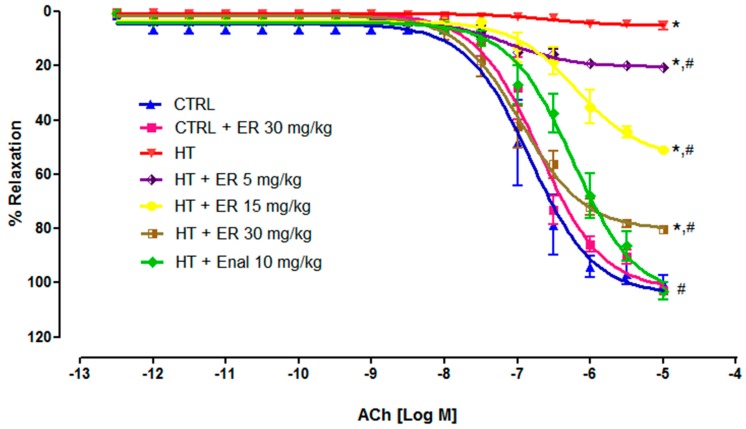
The cumulative concentration-response curves of acetylcholine (ACh) (10^−12.5^–10^−5^ M) on vascular tone of isolated aortic rings from the control group (CTRL), the control group plus ethyl rosmarinate at 30 mg/kg (CTRL + ER 30 mg/kg), the hypertension group (HT), the hypertension group plus ethyl rosmarinate at 5, 15, or 30 mg/kg (HT + ER 5, 15, 30 mg/kg), and the hypertension group plus enalapril at 10 mg/kg (HT + Enal 10 mg/kg). The relaxations were expressed as the percentage decrease in pre-contraction with phenylephrine (PE). Data are expressed as mean ± SEM. *p < 0.05 vs. control group and ^#^*p* < 0.05 vs. hypertension group.

At the end of the experiment, the thoracic aorta was used to verify the efficiency of vascular function. The ability of endothelial cells to induce vasorelaxation was investigated using ACh. Aortic rings were pre-contracted by 1 µM of PE before exposure to ACh cumulatively at concentrations from 3 × 10^−13^ to 3 × 10^−5^ M. The results showed that the aortic rings obtained from the HT group did not respond to ACh. The treatment with ethyl rosmarinate significantly increased the percentage of vasorelaxation with a maximum efficacy (*E_max_*) of 20.64 ± 0.41%, 51.04 ± 0.71%, and 80.43 ± 0.48% for HT + ER groups at concentrations of 5, 15, and 30 mg/kg, respectively. These results are consistent with those shown in the HT + Enal group, in which the tension significantly decreased after exposure to ACh with an *E_max_* of 103.14 ± 3.09% (Figure 2 and Table 2). 

#### 3.2.2. Effect of Ethyl Rosmarinate on Endothelium-Independent Induced Vasorelaxation

**Figure 3:**
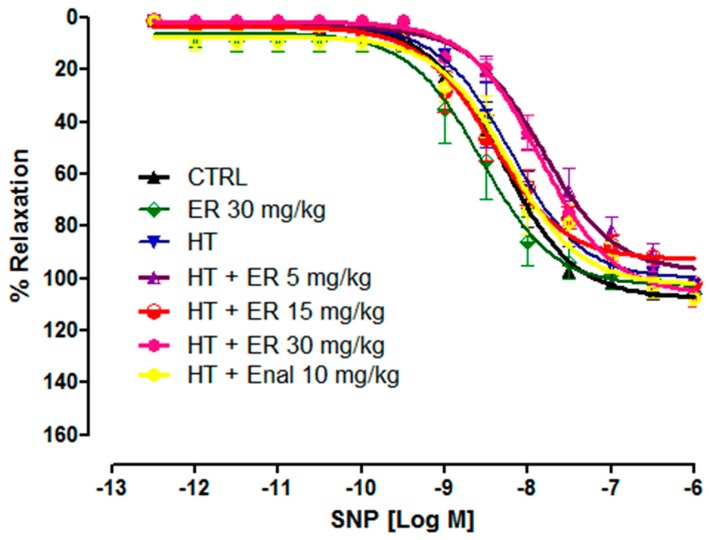
The cumulative concentration-response curves of sodium nitroprusside (SNP) (10^−12.5^–10^−6^ M) on vascular tone of isolated aortic rings from the control group (CTRL), the control group plus ethyl rosmarinate at 30 mg/kg (CTRL + ER 30 mg/kg), the hypertension group (HT), the hypertension group plus ethyl rosmarinate at 5, 15, or 30 mg/kg (HT + ER 5, 15, 30 mg/kg), and the hypertension group plus enalapril 10 mg/kg (HT + Enal 10 mg/kg). The relaxations were expressed as the percentage decrease in pre-contraction with PE. Data are expressed as mean ± SEM.

The ability of aortic rings to carry out endothelium-independent induced vasorelaxation was evaluated by using SNP (from 3 × 10^−12^ to 3 × 10^−6^ M). The results revealed that the tension of all treatments in which pre-contraction by 1 µM of PE had taken place completely decreased after exposure to SNP. This was not significantly different to the CTRL group, as shown in Figure 3 and Table 2. 

#### 3.2.3. Effect of Ethyl Rosmarinate on α_1_-Adrenergic Receptor-Induced Vasoconstriction

**Figure 4:**
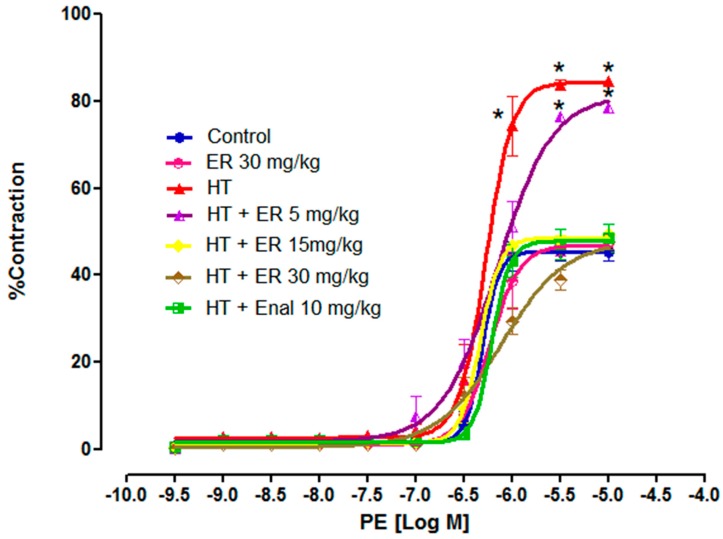
The cumulative concentration-response curves of PE (10^−9.5^–10^−5^ M) on vascular tone of isolated aortic rings from the control group (CTRL), the control group plus ethyl rosmarinate at 30 mg/kg (CTRL + ER 30 mg/kg), the hypertension group (HT), the hypertension group plus ethyl rosmarinate at 5, 15, or 30 mg/kg (HT + ER 5, 15, 30 mg/kg), and the hypertension group plus enalapril 10 mg/kg (HT + Enal 10 mg/kg). Data are expressed as mean ± SEM of the percentage contractions induced by PE. *p < 0.05 vs. Control group.

PE is the specific agonist of the α_1_-adrenergic receptor, which induces the contraction of blood vessels. We found that PE significantly induced vasoconstriction of the aortic rings in the HT and HT + ER (5 mg/kg) groups with *E_max_* of 84.59 ± 0.84% and 78.36 ± 0.29%, respectively. These results showed significant differences in comparison to the CTRL group (*E_max_* = 45.42 ± 2.20%). However, the tension of the aortic rings in the HT + ER (15 and 30 mg/kg ) and enalapril (10 mg/kg) groups showed the same pattern as the CTRL group with *E_max_* of 49.47 ± 1.08%, 47.74 ± 1.72%, and 48.49 ± 3.21%, respectively (Figure 4 and Table 2).

### 3.3. Morphometric Analysis

**Figure 5:**
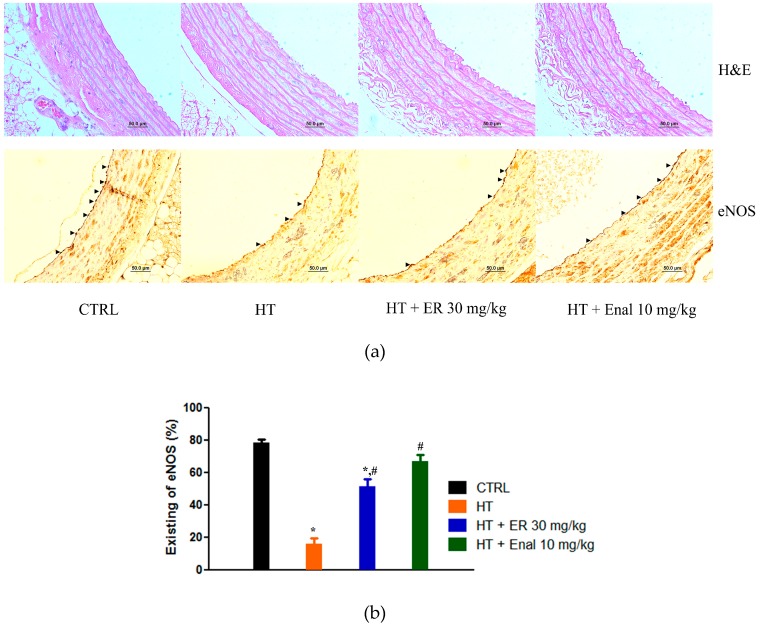
Effect of ethyl rosmarinate on vascular thickening and the expression of endothelial nitric oxide synthase (eNOS) in endothelial cell cytoplasm represented as aortic staining with H&E and eNOS. (**a**) Arrows indicate positive staining of eNOS in endothelial cells. (**b**) The existing eNOS expression measured by immunohistochemistry represented as mean ± SEM. *p < 0.05 vs. control group and ^#^*p* < 0.05 vs. hypertension group.

In the case of the H&E staining, the thickness of the tunica media (TM) in all experimental groups did not show any significant differences (Figure 5). 

### 3.4. Effect of Ethyl Rosmarinate on the Expression of eNOS in Endothelial Cells

Immunohistochemistry showed eNOS staining in the aorta, which was represented as a dark brown color. In the HT group, the expression of eNOS was lower than the CTRL group. In contrast, the expression of eNOS in the HT + ER 30 mg/kg group was higher than the HT group, which was similar to that seen in the HT + Enal group.

### 3.5. Effect of Ethyl Rosmarinate on Vasorelaxation Associated with the eNOS Pathway

**Figure 6:**
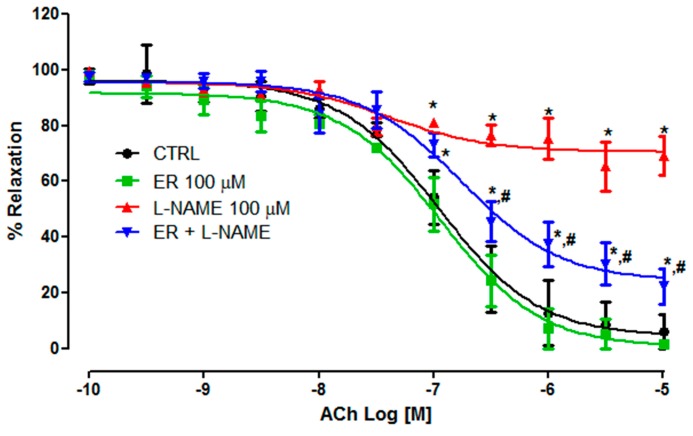
The cumulative concentration-response curves of ACh (10^−10^-10^−5^ M) on vascular tone of endothelium-intact aortic rings of the control group (CTRL), the ethyl rosmarinate 100 µM group (ER 100 µM), the L-NAME 100 µM group (L-NAME 100 µM), and the ethyl rosmarinate 100 µM plus L-NAME 100 µM group (ER + L-NAME). The data are expressed as the percentage decrease in pre-contraction with PE. *p < 0.05 vs. control group and ^#^*p* < 0.05 vs. hypertension group.

The aortic rings were incubated with L-NAME (eNOS inhibitor, 100 μM) or L-NAME plus ethyl rosmarinate (100 µM) before the ACh-induced relaxation. The results revealed that the incubation with L-NAME plus 100 µM ethyl rosmarinate significantly reduced the effect of L-NAME on acetylcholine-induced vasorelaxation (*E_max_* = 22.23 ± 6.31%) in comparison with the L-NAME pretreated group (*E_max_* = 69.24 ± 7.05%) (Figure 6). The results suggest that ethyl rosmarinate could attenuate the effect of L-NAME on acetylcholine-induced relaxation.

## 4. Discussion

This study confirmed that L-NAME induced high blood pressure with endothelial dysfunction. The main finding was that ethyl rosmarinate prevented both the increase in blood pressure and the decrease in the vasorelaxation response to ACh. In addition, the expression of eNOS was higher in the treatment groups while there were no differences in the thickening of the vascular wall between groups. In addition, vascular smooth muscle cells sensitive to epinephrine decreased in the group treated with ethyl rosmarinate and enalapril. This difference was represented by the reduction in the percentage of contraction induced by PE. Furthermore, the vasorelaxation effect of ethyl rosmarinate was attenuated by pretreatment with L-NAME, which indicated its mechanism is related to the eNOS pathway. In addition, ethyl rosmarinate was not found to be toxic, as indicated by the lack of significant change in body weight.

Endothelial dysfunction is one of the critical factors associated with the progression of hypertension. It is characterized by the decrease in NO-induced vasodilation and the lowering of the expression of eNOS in the endothelial cytoplasm [1,3,19]. The use of L-NAME is well established in animal models to mimic hypertension in humans. Prolonged administration of L-NAME disrupts the synthesis of NO, which then increases systemic vasoconstriction and blood pressure [4,20,21]. Our results demonstrated that administration of L-NAME for four weeks led to a progressive increase of SBP above that of the control group. This finding was associated with the decrease in eNOS expression by endothelial cells induced by L-NAME. However, concurrent treatment with ethyl rosmarinate was shown to prevent this alteration. Other studies have shown that the parent compound of ethyl rosmarinate, rosmarinic acid, caused vasodilatory effects regulated through the endothelium-dependent pathway and against endothelial dysfunction through the 5′ adenosine monophosphate-activated protein kinase (AMPK)/eNOS pathway [16,22]. From the results of this study, it can be proposed that ethyl rosmarinate prevented hypertension through the up-regulation of the eNOS signaling pathway. However, the level of NO-induced vasodilation is regulated by several humoral agents and shear stress. The released NO can diffuse into the tunica media and activate the soluble guanylate cyclase (sGC) in vascular smooth muscle cells to cause vasodilation [23,24]. ACh is one of neurotransmitters in the autonomic nervous system, acting through the muscarinic receptors, that induces vasodilation through conduction of the translocation of eNOS, which then increases the level of NO [25,26,27,28]. Therefore, chronic administration of L-NAME leads to a decrease in the ability of ACh to produce NO, which then reduces vasorelaxation. However, the vasorelaxation response to ACh depends on different factors. These factors include the metabolism of NO, the sensitivity of smooth muscle cells to NO, and an imbalance of vasodilator and vasoconstrictor agents [29]. Therefore, the sensitivity of smooth muscle cells to NO was further verified by using SNP, which is a NO donor used to reduce blood pressure in the case of acute hypertension. SNP has also been used in the treatment of aortic valve stenosis, myocardial infarction, and pulmonary hypertension [30,31,32]. 

From our results, prolonged receipt of L-NAME significantly deflated the response curve of ACh-induced vasorelaxation. However, concurrent treatment with ethyl rosmarinate prevented this effect, which increased the response curve of ACh-induced vasorelaxation in a dose-dependent manner. The greater the concentration of ethyl rosmarinate given to rats, the more effective the result for preventing vascular dysfunction. In addition, the response curve of ACh-induced vasorelaxation for ethyl rosmarinate was partially attenuated after pretreatment with L-NAME. This result indicated that ethyl rosmarinate regulated vasorelaxation associated with the eNOS pathway. However, the remaining vasorelaxation effect may be related to its mechanisms through the endothelium-independent pathway [17]. On the other hand, the response curve of SNP-induced vasorelaxation did not show any difference between groups. These results indicated that ethyl rosmarinate promoted vasorelaxation, which may be related to the increase in eNOS which then increases the response to ACh with no alteration in vascular smooth muscle cell integrity. These results are in accordance with previous studies that stated that rosmarinic acid dilated aortic rings with an endothelium-dependent pathway and prevented endothelial cell damage in high-glucose conditions [16,33].

Besides vasodilation, blood vessels are regulated by vasoconstrictor agents, such as endothelin, prostaglandin H_2_, and thromboxane A_2_. Endothelin is one important vasoconstrictor agent that can be stimulated by epinephrine, resulting in an increase in total peripheral resistance (TPR) and blood pressure [34,35]. In cases of hypertension, endothelin is increased, which then increases the sensitivity of smooth muscle cells to epinephrine and results in greater vasoconstriction [36,37,38,39]. Therefore, the percentage of vasoconstriction induced by PE, the sympathomimetic drug that mimics the actions of epinephrine, in the hypertensive group was higher than that observed in the normal group. However, we found that treatment with ethyl rosmarinate significantly decreased this condition, which implies that the sensitivity to epinephrine was reduced by ethyl rosmarinate which then further reduced blood pressure. Normally, PE is a selective α_1_-adrenoceptor agonist that induces vasoconstriction through extracellular Ca^2+^ influx. Our results are consistent with a previous study that showed that ethyl rosmarinate reduced vasoconstriction induced by PE, the regulation thereof being through the endothelium-independent pathway mediated by receptor-operated calcium channel inhibition [17]. 

## 5. Conclusions

From the results of this study, we suggest that ethyl rosmarinate prevented hypertension through enhancing vascular function. The possible mechanisms used by ethyl rosmarinate are the restraining of endothelial dysfunction by increasing the expression of eNOS, which are efficacious effects for hypertensive patients. In addition, ethyl rosmarinate enhanced the decrease in vasoconstrictor sensitivity, which then reduced blood pressure. Furthermore, our previous study demonstrated that ethyl rosmarinate can directly cause vasodilation through vascular smooth muscle cells, which is useful for hypertensive patient that exhibit endothelial dysfunction [17]. Therefore, ethyl rosmarinate is an interesting natural compound that can be used as a dietary supplement or in combination with prescribed medication to reduce the medication dose and incidence of side effects. 

## Figures and Tables

**Table 1:** Effect of ethyl rosmarinate on hemodynamic status in all experimental groups.

Parameters	CTRL	ER (30 mg/kg)	HT	HT + ER (5 mg/kg)	HT + ER (15 mg/kg)	HT + ER (30 mg/kg)	HT + Enal (10 mg/kg)
SBP (mmHg)	114.7 ± 0.5	114.4 ± 0.4	145.8 ± 0.3*	127.9 ± 0.5^#^	124.2 ± 1.0^#^	116.1 ± 0.6^#^	114.2 ± 0.3^#^
Heart rate (beats/min)	429.3 ± 4.9	411.8 ± 7.5	455.3 ± 17.9	430.8 ± 10.0	426.9 ± 4.0	446.1 ± 11.4	412.4 ± 9.1
Body weight (g)	378.3 ± 8.5	406.3 ± 9.2	404.5 ± 5.3	382.8 ± 26.8	394.8 ± 7.9	396.0 ± 12.6	378.3 ± 8.5

Data are expressed as mean ± SEM. (*n* = 6/group) *p < 0.05 vs. control group. ^#^*p* < 0.05 vs. hypertension group. CTRL, control; ER, ethyl rosmarinate; HT, hypertension; HT + ER, hypertension plus ethyl rosmarinate; HT + Enal, hypertension plus enalapril.

**Table 2:** Effect of ethyl rosmarinate on the *E_max_* of ACh- and SNP-induced vasorelaxation and PE-induced contraction.

%*E_max_*	CTRL	ER (30 mg/kg)	HT	HT + ER (5 mg/kg)	HT + ER (15 mg/kg)	HT + ER (30 mg/kg)	HT + Enal (10 mg/kg)
Ach	100.2 ± 3.0	102.9 ± 1.4	5.2 ± 1.5*	20.6 ± 0.4*	51.0 ± 0.7*	80.4 ± 0.5	103.1 ± 3.1
SNP	105.5 ± 1.1	105.7 ± 2.7	102.7 ± 3.3	102.3 ± 1.3	101.7 ± 0.6	106.9 ± 4.5	107.8 ± 2.8
PE	45.4 ± 2.2	47.4 ± 0.6	84.6 ± 0.8*	78.4 ± 0.3*	49.5 ± 1.1	47.7 ± 1.7	48.5 ± 3.2

Data are expressed as mean ± SEM. (*n* = 6/group). *p < 0.05 vs. Control group.
